# Comorbid conditions after allogeneic haematopoietic stem cell transplantation in young adults in Germany—A prospective, multicentre trial

**DOI:** 10.1111/bjh.70738

**Published:** 2026-08-10

**Authors:** Pauline Rocke, Lena Tandetzky, Daniel Wolff, Katharina Egger‐Heidrich, Daniela Heidenreich, Kerstin Schaefer‐Eckart, Eva Wagner‐Drouet, Jochen J. Frietsch, Andreas Hochhaus, Inken Hilgendorf

**Affiliations:** ^1^ Klinik für Innere Medizin II, Hämatologie und Internistische Onkologie Universitätsklinikum Jena Jena Germany; ^2^ Klinik und Poliklinik für Innere Medizin III, Hämatologie und Internistische Onkologie, Universitätsklinikum Regensburg Regensburg Germany; ^3^ Medizinische Klinik und Poliklinik 1, Universitätsklinikum Carl Gustav Carus Dresden Germany; ^4^ DKMS Clinical Trials Unit Dresden Germany; ^5^ III. Medizinische Klinik, Universitätsmedizin Mannheim Mannheim Germany; ^6^ Klinik für Onkologie und Hämatologie, Klinikum Nürnberg Nürnberg Germany; ^7^ III. Medizinische Klinik und Poliklinik, Universitätsmedizin Mainz Mainz Germany; ^8^ Medizinische Klinik und Poliklinik II, Universitätsklinikum Würzburg Würzburg Germany

**Keywords:** comorbidities, graft‐versus‐host disease (GvHD), Post‐transplant Multimorbidity Index (PTMI), stem cell transplantation (SCT), young adults (YA)

## Abstract

Young adults (YA) are particularly affected by comorbid conditions following allogeneic haematopoietic stem cell transplantation (alloHSCT), as they have to live with them for many years. This is the first multicentre prospective study investigating sequelae after alloHSCT in YA in Germany. In total, 107 patients who survived relapse‐free until day 100 after alloHSCT were enrolled. Median patient age was 29.3 years. Chronic graft‐versus‐host disease (cGvHD) occurred in 40.2%. Comorbidities were captured at days 100, 180, 365 and 910 by applying the post‐transplant multimorbidity index (PTMI). Considering all time points, 12.5%–23.5% of patients had no comorbidity, while 39.2%–49.5% had 1–3, 26.3%–33.0% had 4–6 and 5.7%–8.4% had >6 PTMI‐defined comorbidities respectively. The most prevalent PTMI‐based comorbidities were iron overload, hypogonadism, arterial hypertension, infections, hepatic dysfunction and osteopenia/osteoporosis. However, the spectrum of PTMI‐recorded comorbidities and those concurrently self‐reported by patients differed substantially. Factors linked to a higher PTMI were total body irradiation and active cGvHD. Investigating single PTMI‐defined comorbidities, we found significant associations of osteopenia/osteoporosis with cGvHD, ongoing immunosuppression and hypogonadism, which was more common among female survivors. These results provide a detailed analysis of the occurrence of a vast spectrum of PTMI‐defined comorbidities in YA after alloHSCT.

## INTRODUCTION

Adolescents and young adult (AYA) patients are those diagnosed between 15 and 39 years from cancer,^1^ hence those aged 18–39 years are described as young adults (YA). YA represent a minority among the population of survivors of allogeneic haematopoietic stem cell transplantation (alloHSCT).^2^ They are particularly vulnerable to profound changes to their lives caused by diagnosis and treatment. Despite increasing survival rates for YA after alloHSCT,^2^ recent studies still show a poorer outcome for AYA compared to children.^3^ Contributing factors include differences in disease biology and treatment protocols, as well as poorer adherence to therapy and follow‐up care.^4^, ^5^, ^6^ Furthermore, due to their young age, aggressive treatment protocols and high cure rates, YA are particularly affected by long‐term sequelae.^7^ Recipients of alloHSCT experience a variety of late effects that can affect most organ systems: 66.4% report at least one chronic health condition and 18.3% report a severe or life‐threatening condition.^8^ Especially chronic graft‐versus‐host disease (cGvHD) contributes significantly to the increased risk of comorbid conditions.^8^, ^9^ As those late effects are associated with a high morbidity and mortality, their prevention and treatment gain significance. While sequelae following alloHSCT in paediatric and elderly patients have been investigated in several studies,^8^, ^10^, ^11^ there is a lack of publications inquiring long‐term outcomes in YA.^9^ Recently, the Post‐transplant Multimorbidity Index (PTMI), a new measure to capture comorbidity in survivors, has been developed and validated.^12^ The PTMI demonstrated strong measurement properties and, compared to the Charlson Comorbidity Index^13^ and Hematopoietic Cell Transplantation‐Specific Comorbidity Index,^14^ captured a wider range of sequelae. The score also correlated with the severity of cGvHD, the quality of life (QoL) and physical functioning.^12^

This is the first multicentre prospective study in which the PTMI was used to record comorbidities in YA in a prospective setting in order to investigate sequelae after alloHSCT in Germany. The specific objectives of this trial were to quantify the burden of PTMI‐based comorbidity in YA after alloHSCT, to demonstrate the manifestation of late effects over the course of time and to identify risk factors associated with increased comorbidity as well as specific late effects.

## SUBJECTS AND METHODS

### Design and setting

This prospective, multicentre, non‐interventional trial among six transplant centres in Germany was approved by the local ethics committee of Jena University Hospital (Reg.‐Nr. 4670‐01/16). All patients transplanted consecutively that fitted the inclusion criteria were invited to participate. Eligibility criteria included aged 18–39 years at date of alloHSCT, relapse‐free survival until inclusion, point of inclusion to the trial at day (d) 100 (±21 days) after alloHSCT and provided signed informed consent. Patients that withdrew their consent, had a calculated life expectancy lower than 6 months, were unable to understand and fill out the forms or suffered from relapse of the underlying malignancy, were excluded. Between 2016 and 2022, 109 patients were enrolled. Two patients were excluded because of early relapse, leading to 107 patients in the final analysis.

### Data collection

Patient characteristics were entered into the ‘Computer‐based Health‐Evaluation‐System (CHES) – GvHD‐Register’ (Innsbruck, Austria). Graft‐versus‐host disease (GvHD) status, ‐treatment, immunosuppression and comorbidities were documented at d100 (±21), 180 (±21), 365 (±21) and 910 (±60). Information regarding remission and survival were collected at each date.

### Measures

The underlying definition of comorbidity and the calculation of the PTMI were applied as previously published.^12^ In addition, at d365 and d910, patients completed several self‐report questionnaires including the occurrence of adverse medical conditions. This list of comorbidities included 28 items, not identical to the comorbidities contained in the PTMI (see Supplementary Information S1).

### Statistical analysis

Descriptive statistics were calculated for demographic data and transplantation factors. To examine the determinants of the PTMI, Mann–Whitney *U*‐test was applied. Pearson's chi‐squared test or Fisher's exact test were used to examine factors influencing single comorbidities. Effect size was calculated using Pearson's correlation (‘r’). The effect sizes were categorized as small (0.1–<0.3), medium (0.3–<0.5) or large (>0.5). To investigate whether the occurrence of comorbidities differed across the assessed time points, a distribution analysis was conducted using the two‐factor Friedman test for related samples, and the significance level was adjusted for multiple comparisons using the Bonferroni correction. A *p*‐value of <0.05 was considered statistically significant. SPSS Statistics 29 (IBM, Chicago) was used for all analyses.

## RESULTS

### Study population

In total, 107 patients with a median age of 29.3 (range 18.6–39.9) years suffering from acute myeloid (38.3%) or lymphatic (23.4%) leukaemia, followed by lymphoma (12.1%) and non‐malignant diseases (11.2%), participated. The majority of patients received peripheral blood stem cells (81.3%) from an unrelated donor (78.5%) after myeloablative conditioning (MAC, 64.5%) and/or total body irradiation (TBI, 55.1%). At time of alloHSCT, 64.5% of patients were in remission. At the prespecified time points, 79.4% (d100), 43.9% (d180), 32.6% (d365) and 11.8% (d910) received an immunosuppressive treatment respectively. In total, 47.7% of patients had developed aGvHD and 40.2% cGvHD, which was active at d100/180/365/910 in 15%/27.3%/21.5%/11.3% of patients and showed a mild presentation in 68.8%/55.6%/50.0%/50.0% respectively. Overall survival at d910 was 84.8%. The patient characteristics are displayed in Table 1.

**TABLE 1:** Patient, disease and transplantation characteristics of the study population.

Characteristics	Frequency (%)
Number of patients	107
Per centre	
Jena	39 (36.4)
Regensburg	29 (27.1)
Dresden	20 (18.7)
Mainz	9 (8.4)
Nürnberg	6 (5.6)
Mannheim	4 (3.7)
Recipient sex	
Male	67 (62.6)
Female	40 (37.4)
Age at alloHSCT in years (range)	29.3 (18.6–39.9)
Diagnosis	
*Acute leukaemia*	66 (61.7)
Acute myeloid leukaemia	41 (38.3)
Acute lymphatic leukaemia	25 (23.4)
*Lymphoma*	13 (12.1)
Non‐Hodgkin‐Lymphoma	9 (8.4)
Hodgkin‐Lymphoma	3 (2,8)
Chronic lymphatic leukaemia	1 (0.9)
*Non‐malignant*	12 (11.2)
*Others*	16 (15.0)
MDS/MPN	6 (5.6)
Chronic myeloid leukaemia	6 (5.6)
Multiple myeloma	2 (1.9)
Acute leukaemia, unspecified	2 (1.9)
Time from diagnosis to alloHSCT (median)	6 months
Previous treatments	
Allogeneic HSCT (number: one, two)	7 (6.5) (6 (5.6), 1 (0.9))
Autologous HSCT	6 (5.6)
Conditioning regimen	
Myeloablative	69 (64.5)
Non‐myeloablative	38 (35.5)
Total body irradiation	59 (55.1)
Dose in Gray (%)	2 (13.6), 8 (23.7), 12 (62.7)
Graft source	
Peripheral blood	87 (81.3)
Bone marrow	20 (18.7)
Donor type	
Related	23 (21.5)
Unrelated	84 (78.5)
Disease status at alloHSCT	
Remission	69 (64.5)
Complete remission	61 (57.0)
Partial remission	8 (7.5)
Progress or relapse	15 (14.0)
Other	11 (10.3)
Unknown	12 (11.2)
Karnofsky status at alloHSCT	
90–100	91 (85.0)
70–80	12 (11.2)
Acute GvHD	51 (47.7)
Chronic GvHD	43 (40.2)
Severity at d100, d180, d365, d910 (%)	
Mild	68.8, 55.6, 50.0, 50.0
Moderate	31.2, 44.4, 30.0, 16.7
Severe	0.0, 0.0, 20.0, 33.3

Abbreviations: alloHSCT, allogeneic haematopoietic stem cell transplantation; d, day; GvHD, graft‐versus‐host disease; MDS/MPN, myelodysplastic/Myeloproliferative neoplasm.

### Documentation status

A fully documented PTMI was available at d100/180/365/910 for 95/88/82/51 patients respectively. Reasons for exclusion or dropout from the study were relapse or death of 5/11/22 patients and a lost to follow‐up of 2/2/22 patients at d180/365/910 respectively. The self‐report questionnaire of comorbidities was completed by 89 patients at d365 and 29 patients at d910.

### The PTMI

#### Burden of PTMI‐defined comorbidity

Considering all time points, 12.5%–23.5% of patients had no comorbidities, while 39.2%–49.5% had 1–3, 26.3%–33.0% had 4–6 and 5.7%–8.4% suffered from >6 PTMI‐defined comorbidities. Median PTMI remained stable at 3 sequelae per patient with a slight decrease at d365 (median 2, 5). At least one PTMI‐defined comorbidity was present at d100/180/365/910 in 84.2%/87.5%/85.4%/76.5% of patients respectively.

#### Frequency of comorbidities

The distribution of the comorbidities by organ systems differed across the individual time points. Whereas at d100 37.9% of patients suffered from at least one cardiovascular sequelae, only 17.6% were affected at d910. Gastrointestinal conditions were present in 52.6% of patients at d100 and declined to 17.6% at d910 (*p* < 0.001). Endocrine, pulmonary and neuropsychiatric sequelae showed a slight incline over the course of time. Conditions of the skeletal system rose significantly from 10.5% of patients affected at d100 to 31.4% at d910 (*p* < 0.001). The percentage of patients with at least one PTMI‐defined comorbidity of the affected organ system is illustrated in Figure 1.

**FIGURE 1:**
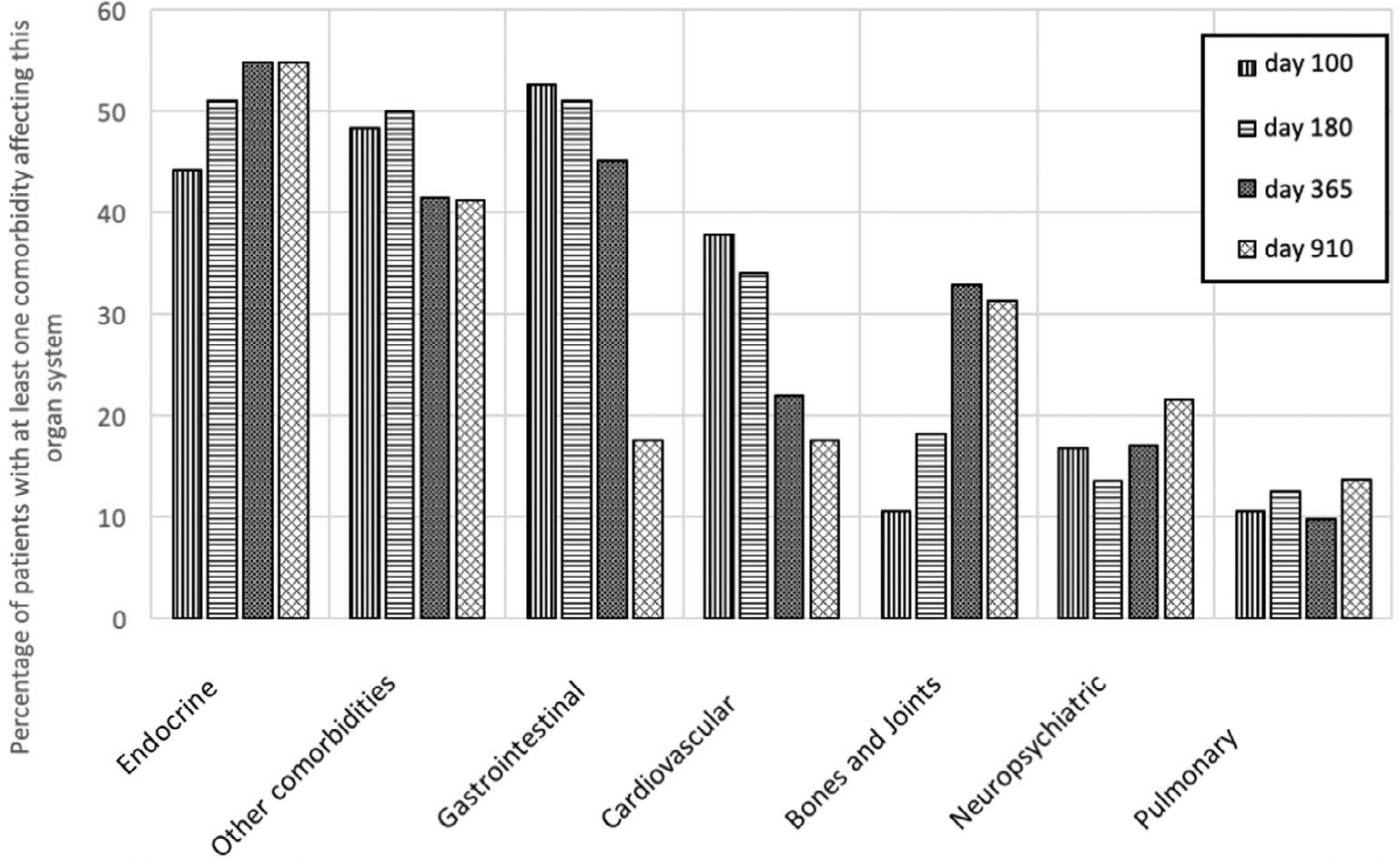
Patients with at least one PTMI‐defined comorbidity affecting this organ system.

Looking into the most prevalent single PTMI‐defined comorbidities, iron overload, defined as an elevated serum ferritin >1000 μg/mL, was the most common late effect in the first year, but decreased until day 910 (*p* < 0,001). The occurrence of osteopenia/osteoporosis rose significantly post‐alloHSCT (*p* = 0.002). Moreover, a considerable number of YA developed avascular necrosis, in some cases severe enough to require joint replacement, or osteoarthritis as a further orthopaedic sequela by d910.

The prevalence of hepatic dysfunction declined over time (*p* = 0.056). Considering cardiovascular risk factors, the prevalence of dyslipidaemia inclined steadily (*p* = 0.014), while arterial hypertension declined non‐significantly until d910 (*p* = 0.459). Regarding endocrinopathies, an increase of hypogonadism was notable (*p* = 0.277). In addition, hypothyroidism remained among the most common comorbidities at each time point. Even though smoking was rare, up to 7.4% of YA reported on nicotine abuse. At each point, about one quarter of the YA suffered from infections. Psychiatric disorders requiring treatment were reported at each time point and exceeded the 10% threshold at d365, mainly caused by anxiety and panic disorders. Figure 2 illustrates the occurrence of the most common single comorbidities over the course of time. Frequencies of comorbidities captured by the PTMI are shown in Table 2, with detailed descriptions and underlying definitions provided in Table S3.

**FIGURE 2:**
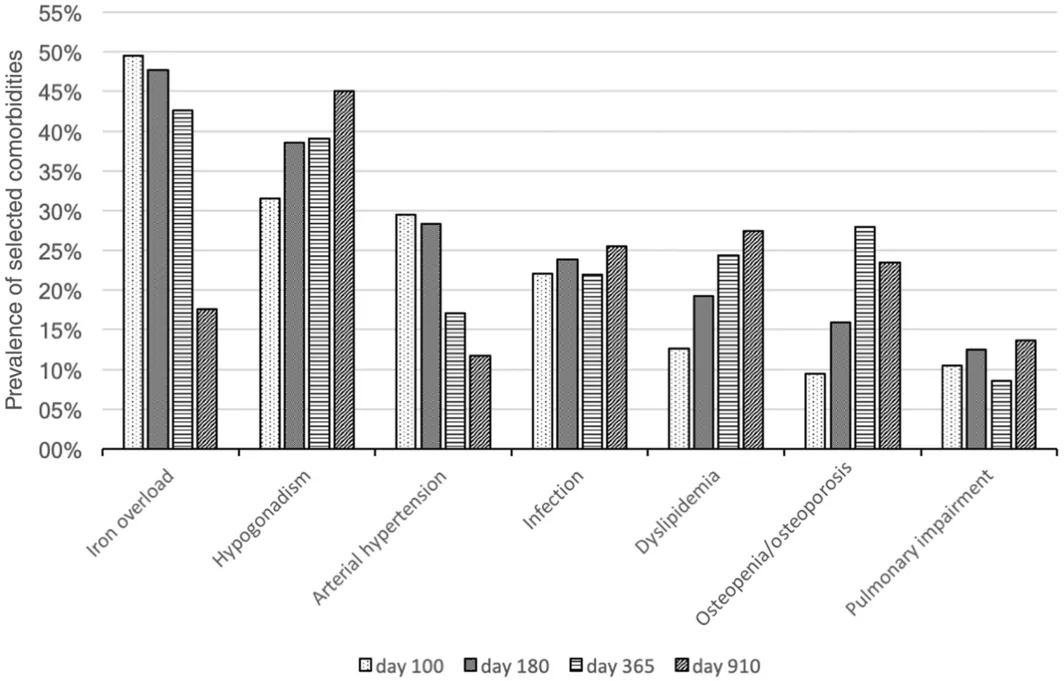
Prevalence of selected common PTMI‐defined comorbidities following allogeneic haematopoietic stem cell transplantation.

**TABLE 2:** Frequency of comorbidities present in the studied cohort as captured by the PTMI.

Comorbidity (*n* = number; % = frequency)	Day 100 (*n* = 95)		Day 180 (*n* = 88)		Day 365 (*n* = 82)		Day 910 (*n* = 51)	
	*n*	%	*n*	%	*n*	%	*n*	%
1. Cardiovascular								
Arrhythmia	2	2.1%	1	1.1%	0	0.0%	1	2.0%
Heart valve disease	1	1.1%	1	1.1%	1	1.2%	1	2.0%
Congestive heart failure	1	1.1%	2	2.3%	4	4.9%	1	2.0%
Cerebrovascular disease	2	2.1%	1	1.1%	2	2.4%	0	0.0%
Venous thrombosis	9	9.5%	7	8.0%	2	2.4%	0	0.0%
Compensated hypertension	27	28.4%	25	28.4%	14	17.1%	6	11.8%
Uncontrolled hypertension	1	1.1%	0	0.0%	0	0.0%	0	0.0%
2. Gastrointestinal								
Peptic ulcer/hernia/reflux	2	2.1%	1	1.1%	0	0.0%	0	0.0%
Mild hepatic	9	9.5%	8	9.1%	5	6.1%	2	3.9%
Moderate/severe hepatic	2	2.1%	3	3.4%	1	1.2%	0	0.0%
Iron overload	47	49.5%	42	47.7%	35	42.7%	9	17.6%
Inflammatory bowel disease	1	1.1%	0	0.0%	0	0.0%	0	0.0%
3. Pulmonary								
Moderate pulmonary	8	8.4%	11	12.5%	6	7.3%	6	11.8%
Severe pulmonary	2	2.1%	0	0.0%	1	1.2%	1	2.0%
Pulmonary hypertension	0	0.0%	0	0.0%	0	0.0%	1	2.0%
Asthma	0	0.0%	1	1.1%	1	1.2%	1	2.0%
4. Endocrine								
Hyperglycaemia	0	0.0%	1	1.1%	1	1.2%	1	2.0%
Diabetes	5	5.3%	2	2.3%	1	1.2%	1	2.0%
Hypothyroidism	8	8.4%	10	11.4%	13	15.9%	5	9.8%
Hyperthyroidism	0	0.0%	3	3.4%	0	0.0%	1	2.0%
Hypogonadism	30	31.6%	34	38.6%	32	39.0%	23	45.1%
Adrenal insufficiency	3	3.2%	2	2.3%	2	2.4%	1	2.0%
Hypertriglyceridaemia	7	7.4%	7	8.0%	14	17.1%	7	13.7%
Hypercholesterolaemia	8	8.4%	14	15.9%	13	15.9%	11	21.6%
5. Neuropsychiatric								
Psychiatric disturbance‐Depression	3	3.2%	1	1.1%	4	4.9%	0	0.0%
Psychiatric disturbance‐Anxiety or panic disorder	5	5.3%	4	4.5%	5	6.1%	6	11.8%
Neurological disease	9	9.5%	8	9.1%	5	6.1%	5	9.8%
Visual impairment	2	2.1%	1	1.1%	1	1.2%	3	5.9%
Hearing impairment	1	1.1%	0	0.0%	1	1.2%	0	0.0%
6. Bone/Joint								
Osteoarthritis	0	0.0%	1	1.1%	0	0.0%	5	9.8%
Degenerative disc disease (spinal stenosis or severe chronic back pain)	0	0.0%	0	0.0%	1	1.2%	0	0.0%
Avascular necrosis	3	3.2%	3	3.4%	4	4.9%	7	13.7%
Osteopenia/Osteoporosis	9	9.5%	14	15.9%	23	28.0%	12	23.5%
Rheumatological	0	0.0%	1	1.1%	1	1.2%	1	2.0%
7. Other comorbidities								
Mild renal	7	7.4%	5	5.7%	6	7.3%	5	9.8%
Moderate/severe renal	0	0.0%	0	0.0%	2	2.4%	1	2.0%
Proteinuria	1	1.1%	1	1.1%	2	2.4%	1	2.0%
Prior solid malignancy	4	4.2%	4	4.5%	5	6.1%	3	5.9%
Solid malignancy	2	2.1%	2	2.3%	1	1.2%	0	0.0%
Post‐transplant lymphoproliferative disorder	2	2.1%	0	0.0%	0	0.0%	0	0.0%
Nicotine abuse	7	7.4%	6	6.8%	5	6.1%	1	2.0%
Mild infection	15	15.8%	12	13.6%	15	18.3%	10	19.6%
Moderate infection	5	5.3%	8	9.1%	2	2.4%	2	3.9%
Severe infection	1	1.1%	1	1.1%	1	1.2%	1	2.0%
Obesity	8	8.4%	6	6.8%	3	3.7%	3	5.9%
Severe obesity	2	2.1%	3	3.4%	2	2.4%	1	2.0%
Underweight	11	11.6%	9	10.2%	4	4.9%	1	2.0%
Other comorbid conditions (patients affected)	5 (5)	5.3%	6 (6)	6.8%	9 (6)	8.5%	8 (6)	15.7%

Abbreviation: PTMI, Post‐transplant Multimorbidity Index.

### Determinants

Investigating the determinants of the PTMI, at d100, non‐MAC was associated with a higher score (*p* = 0.005, *r* = 0.29, median PTMI 2.0 vs. 4.0), while no significant correlation was found for other time points. TBI was linked to a higher PTMI‐score at d910 (*p* = 0.013, *r* = 0.34, median PTMI 3.5 vs. 1.0), but not on d100, 180 and 365. An active cGvHD came along with increased PTMI‐defined comorbidity only on d100 (*p* = 0.02, *r* = 0.24, median PTMI 4.0 vs. 2.5), while the severity of the cGvHD showed no influence on the PTMI. Compared with patients who had received only one alloHSCT so far, patients with more than one alloHSCT obtained a median PTMI on d910 that was twice as high (*p* = 0.008, *r* = 0.37, median PTMI 3.0 vs. 6.0). No significant association was found for the sex of the patient, the donor type (related/unrelated), aGvHD, the disease status at alloHSCT and an ongoing immunosuppressive treatment.

Subsequently, the determinants of selected single comorbidities were explored. The influencing factors selected were relations already established in the literature. We found significant associations of osteopenia/osteoporosis with cGvHD (d910, *p* = 0.022), ongoing immunosuppression (d910, *p* = 0.021) and hypogonadism (d100, *p* = 0.017), but not with the sex or TBI. Patients with cGvHD (d180, *p* = 0.021) or active immunosuppressive treatment (d365, *p* = 0.02) showed higher rates of avascular necrosis, while the sex or TBI showed no influence. Hypogonadism was more common among female patients (d100/180/365/910, *p* = 0.010/0.008/0.003/0.044) and among patients with cGvHD (d100, *p* = 0.001), but was not associated with TBI, MAC or immunosuppressive treatment. Iron overload was associated with liver dysfunction (d100/180/910, *p* = 0.023/0.016/0.002), while no correlation was found for cGvHD or immunosuppressive treatment. Patients with nicotine abuse were more likely to suffer from infections (d100, *p* = 0.035) and a pulmonary impairment correlated with infections as a coexisting sequela (d180, *p* = 0.007). No association was found for pulmonary impairment with TBI, MAC and immunosuppressive treatment. Patients who received non‐MAC exhibited higher rates of renal failure at d100 (*p* = 0.047) and d180 (*p* = 0.042). We found an association of hypertension with renal impairment at d365 (*p* = 0.026) and d910 (*p* = 0.017). No correlation was observed for TBI, cGvHD and immunosuppressive treatment. Neurological conditions were more common among patients who had received non‐MAC at d100 (*p* = 0.011) but did not correlate with TBI, cGvHD or immunosuppressive treatment. For psychiatric disorders, no significant difference between male and female patients could be identified. All correlations are displayed in Table S2.

### Comparison with self‐reported comorbidities

Similar to the rates detected by the PTMI, 89.9% of patients self‐reported at least one sequela at d365. However, the spectrum of comorbidities differs considerably: While neurological conditions as detected by the PTMI were relatively rare (6.1%–9.8%), nearly half of patients at d365 (48.3%) and three quarter at d910 (72.4%) reported at least one neurological issue, with concentration difficulties and cognitive impairment being the most reported. The rates of dyspnoea and deterioration of vision, both reported by around one‐third of patients, exceeded the rates of pulmonary (8.5%–13.7%) and visual impairments (1.1%–5.9%) according to the PTMI noticeably. Additionally, 29.2%–41.4% reported ophthalmological problems due to dry eyes. Conversely, the PTMI captured more cases of osteoporosis, osteonecrosis, arterial hypertension, iron overload and kidney diseases. Hepatitis was self‐reported by 4 patients at d365 and 3 patients at d910, while no cases were documented within the PTMI. In total, 39.0% (d365) and 45.1% (d910) of patients were diagnosed with hypogonadism, but only 24.7% and 27.6% reported receiving hormone replacement therapy on the respective time points. The detailed prevalence data of the patient‐reported comorbidities are provided in the Table 3.

**TABLE 3:** Percentage of patients with at least one disease within the respective organ system (patient‐reported).

	d365			d910			*p*‐value^a^
	*N*	%	Missing (*n*)	*n*	%	Missing (*n*)	
Total	89	100.0	0	29	100.0		
Disorders of the nervous system	43	48.3		21	72.4		**<0.001**
Concentration problems	31	34.8	1	11	37.9		
Memory problems	28	31.5	1	17	58.6		
Tingling or numbness	16	18.0	1	9	31.0		
Metabolic diseases	39	43.8		13	44.8		0.791
Hormone replacement therapy	22	24.7	2	8	27.6		
Hypercholesterolaemia	14	15.7	2	7	24.1		
Hypothyroidism	10	11.2	2	2	6.9	1	
Diabetes mellitus	3	3.4	1	1	3.4		
Hyperthyroidism	2	2.2	1	1	3.4	1	
Eye diseases	39	43.8		17	58.6		0.180
Dry eyes	26	29.2	5	12	41.4	1	
Deterioration of vision	24	27.0		9	31.0	2	
Lens opacity	3	3.4	3	3	10.3	1	
Diseases of the gastrointestinal tract	33	37.1		6	20.7		**<0.001**
Iron overload	19	21.3	2	6	20.7		
Hepatitis	4	4.5		3	10.3		
Other^b^	13	14.6		3	10.3		
Respiratory diseases	31	34.8		16	55.2		**0.031**
Dyspnoea on heavy exertion	25	28.1	5	8	27.6	3	
Dyspnoea on mild exertion	6	6.7	6	2	6.9	1	
Dyspnoea at rest	0	0.0	8	0	0.0	2	
Frequent infections	9	10.1	2	9	31.0	1	
Cardiovascular diseases	20	22.5		9	31.0		0.180
Hypertension	12	13.5	2	3	10.3		
Myocardial dysfunction	4	4.5	2	2	6.9		
Valvular heart disease	3	3.4	9	0	0.0	3	
Stroke	2	2.2	2	1	3.4		
Arrhythmias	2	2.2	3	3	10.3		
Myocardial infarction	0	0.0	2	0	0.0		
Diseases of the skeletal system	13	14.6		7	24.1		0.109
Osteoporosis	12	13.5	5	6	20.7	1	
Osteonecrosis	0	0.0	5	3	10.3		
Artificial joint replacement	2	2.2	1	2	6.9		
Kidney diseases	4	4.5		0	0.0		
Further cancer after HSCT	4	4.5	1	2	6.9		
Other illnesses^c^	33	37.1	4	12	41.4		0.832

Abbreviations: GvHD, graft‐versus‐host disease; HSCT, haematopoietic stem cell transplantation.

^a^ *p*‐value refer to comparisons between d365 and d910 (*n* = 29). For kidney diseases and further cancer, *p*‐values could not be calculated due to small group sizes. Statistically significant results (*p* < 0.05) are shown in bold.

^b^ Most frequently mentioned: diarrhoea, GvHD, gastritis.

^c^ Most frequently mentioned: pain, fatigue, dry skin and mucosal membranes.

## DISCUSSION

YA represent a sensitive population among alloHSCT recipients, deserving particular attention but still being understudied in research. However, knowledge about late effects after alloHSCT is essential to establish an adequate follow‐up care for YA that considers their specific needs. Addressing this gap, we used a multicentre, prospective approach to examine sequalae after alloHSCT in YA in Germany. Compared with the validation trial of the PTMI, we observed a higher number of patients with 1–3 and 4–6 comorbidities despite the younger age and the lower proportion of patients with cGvHD, whereas the rate of multimorbid patients was slightly lower.^12^ In comparison with other studies, the patients overall exhibited a higher burden of PTMI‐defined comorbidity. In a questionnaire‐based survey, 71% of patients reported at least one sequela, although hypogonadism as one of the most common chronic comorbidities was not included.^8^ In said assessment, the number and severity of late effects progressively increased steadily. However, we could not demonstrate this effect, likely due to the comparatively short follow‐up. Other studies reported chronic conditions in 19%–79% of HSCT‐recipients.^9^, ^11^, ^15^ In line with previous studies, cGvHD was identified as a primary predictor of increased PTMI‐defined comorbidity. TBI also displayed a risk factor, while the literature provides mixed evidence regarding this association.^8^, ^9^, ^15^, ^16^ The observed association between non‐MAC and increased sequelae can be explained by reversed causality: They were more likely to receive this type of conditioning because of pre‐existing comorbidities.

AlloHSCT is associated with premature cardiovascular disease and adverse events.^17^, ^18^ These findings could not be reproduced in our data, most likely caused by the relatively short follow‐up period and a health‐conscious young age group. Nevertheless, YA exhibited a considerable higher prevalence of cardiovascular risk factors compared with the general population, particularly arterial hypertension,^19^ an established side effect of immunosuppressives and dyslipidaemia.^20^ Consistent detecting of these factors as well as preventive and therapeutic interventions are therefore essential. Lifestyle‐related factors also contribute to the increased cardiovascular risk. While the elevated prevalence of obesity reported in other publications^21^, ^22^ was not observed in our data, a notable proportion of YA engaged in harmful health behaviour through nicotine abuse. Although this behaviour, consistent with another study, occurred less frequently than in the general population,^23^ smoking requires particular attention and patient education, as it represents a modifiable risk factor both for cardiovascular disease and for secondary malignancies.

Iron overload was common and is most plausible linked to transfusions,^24^ while the decrease observed at d910 is attributable to phlebotomy. Pulmonary conditions were less frequent among YA compared with older patients.^12^, ^25^ In line with earlier studies,^26^, ^27^ hypogonadism represented the most significant endocrine late effect among the YA patients, affecting two‐thirds of women and one‐third of men even 30 months after alloHSCT. This emphasizes the importance of hormone replacement after alloHSCT. Neurological sequelae are commonly caused by neurotoxic agents or cGvHD.^28^, ^29^ Consistent with previous publications, depression requiring treatment was rare in terms of psychiatric comorbidities,^30^, ^31^ whereas anxiety disorders were more common. Compared to elderly patients, AYA tend to worry more about their future, treatment factors and emotional role functioning.^32^ Close attention should be paid to bone diseases, as their prevalence increases steadily following alloHSCT. Despite a presumably healthy bone metabolism prior to transplantation, YA patients showed high rates of bone diseases comparable to elderly patients.^33^, ^34^ Kidney injury was scarce among YA patients and consistent with other studies, arterial hypertension emerged as a risk factor.^35^, ^36^ Divergent definitions of renal impairment complicate statements regarding incidence. The documented infections were mostly mild and did not require inpatient hospital care. Although fewer patients were receiving immunosuppressive treatment over time, infection rates did not decrease, most likely attributable to the prolonged immune reconstitution after alloHSCT.^37^

The risk of secondary neoplasms rises continuously with time after alloHSCT. During the follow‐up‐period, two YA developed a secondary neoplasm. Furthermore, five YA already had a solid tumour prior to alloHSCT, and two cases of post‐transplant lymphoproliferative disorder were registered. These findings align plausibly with previous studies^9^, ^38^ and underline the necessity of lifelong, regular participation in cancer screening.

Chronic GvHD is the most significant long‐term complication and is known to increase the risk of several other comorbidities.^12^, ^39^ Although its prevalence among the YA patients was similar as reported in other cohorts,^40^, ^41^ it appeared to follow a mostly favourable course within the relatively short follow‐up period.

Limitations of the study are the relatively small sample size particularly regarding the PTMI‐defined comorbidity analysis. However, this must be evaluated in the context of the limited total number of patients transplanted in young adulthood^2^ who also had to meet the inclusion criteria of relapse‐free survival at d100 post‐alloHSCT. Moreover, no information was available regarding pre‐existing comorbidities. Therefore, comorbidities arising de novo between alloHSCT and the first assessment cannot be distinguished from pre‐existing conditions. Thus, conclusions regarding causal relations between comorbidities and the transplantation process can only be drawn to a limited extent.

In summary, our findings demonstrate that late effects after alloHSCT are widespread also among YA, in whom lower morbidity and fewer pre‐existing comorbidities would be expected compared with elderly patients. The PTMI appears to be a suitable measure for capturing comorbidities after alloHSCT. However, comparison with the questionnaire‐based sequelae self‐assessment in the same cohort showed that, beyond objectively measurable late effects diagnosed by instrumental and laboratory diagnostics, patients experience a substantial burden of symptoms that are less easily objectifiable, but can still impair QoL. On the other hand, some organ impairments are reported less frequently by patients than documented by medical staff, suggesting an information gap on the patient side. This may reflect that not all diagnostic findings were consistently discussed with patients.

Based on these findings, existing follow‐up‐concepts can now be optimized to better meet the specific needs of this age group. Due to a high loss‐to‐follow‐up rate by d910, this analysis primarily allows conclusions regarding the first year after alloHSCT. As extended follow‐up (5 and 10 years) is planned, this article presents the initial findings. Regardless, further trials covering longer follow‐up periods as well as studies specifically designed to evaluate targeted interventions and their impact on post‐transplant outcomes are desirable to obtain more detailed insights into the post‐transplant course.

## AUTHOR CONTRIBUTIONS

**Pauline Rocke:** Investigation; writing – original draft; data curation; formal analysis; visualization. **Kerstin Schaefer‐Eckart:** Writing – review and editing; data curation. **Lena Tandetzky:** Writing – review and editing; formal analysis; data curation. **Daniela Heidenreich:** Writing – review and editing; data curation. **Andreas Hochhaus:** Writing – review and editing; conceptualization; methodology. **Katharina Egger‐Heidrich:** Writing – review and editing; data curation. **Daniel Wolff:** Writing – review and editing; conceptualization; methodology; data curation; supervision; project administration; funding acquisition. **Inken Hilgendorf:** Conceptualization; investigation; writing – original draft; supervision; data curation; methodology; project administration; funding acquisition; visualization. **Jochen J. Frietsch:** Writing – review and editing. **Eva Wagner‐Drouet:** Writing – review and editing; data curation.

## CONFLICT OF INTEREST STATEMENT

Inken Hilgendorf and Daniel Wolff received research funding from the H.W. Hector Foundation. Inken Hilgendorf is chairperson of the board of trustees of the German Foundation for Young Adults with Cancer and received honoraria from AbbVie, Alexion, Medac, Novartis, Sanofi and Takeda. She has received compensation as a member of the scientific advisory board of Mundipharma. Daniel Wolff received honoraria from Novartis, Sanofi, Incyte, Therakos, Behring and research support from Novartis. Katharina Egger‐Heidrich received travel support from Incyte, Johnson&Johnson, Servier and Sanofi, honoraria from Bristol Myers Squibb and compensation as a member of the scientific advisory board from Sanofi, Beigene and Takeda.

## Supporting information


**Table S1.** Questionnaire of patient‐reported comorbidities.

**Table S2.** Determinants of single PTMI‐defined comorbidities.

**Table S3.** Frequency of comorbidities captured by the PTMI including the applied definition.

## Data Availability

The datasets generated during and/or analysed during the current study are available from the corresponding author on reasonable request.

## References

[1] Coccia PF , Pappo AS , Beaupin L , Borges VF , Borinstein SC , Chugh R , et al. Adolescent and young adult oncology, version 2.2018, NCCN clinical practice guidelines in oncology. J Natl Compr Canc Netw. 2018;16(1):66–97. 10.6004/jnccn.2018.0001 29295883

[2] Frietsch JJ , Flossdorf S , Beck JF , Kroger N , Fleischhauer K , Dreger P , et al. Outcomes after allogeneic haematopoietic stem cell transplantation in young adults in Germany. Br J Haematol. 2022;201:308–318. 10.1111/bjh.18610 36573337

[3] Trama A , Geerdes EE , Demuru E , De Angelis R , Karim‐Kos HE , Troussard X , et al. Survival of European children, adolescents and young adults diagnosed with haematological malignancies in the period 2000‐2013: results from EUROCARE‐6, a population‐based study. Eur J Cancer. 2025;222:115336. 10.1016/j.ejca.2025.115336 40334304

[4] Coccia PF . Overview of adolescent and young adult oncology. J Oncol Pract. 2019;15(5):235–237. 10.1200/JOP.19.00075 31009282

[5] Miyamura K , Yamashita T , Atsuta Y , Ichinohe T , Kato K , Uchida N , et al. High probability of follow‐up termination among AYA survivors after allogeneic hematopoietic cell transplantation. Blood Adv. 2019;3(3):397–405. 10.1182/bloodadvances.2018026039 PMC6373751 30728138

[6] Tewari P , Franklin AR , Tarek N , Askins MA , Mofield S , Kebriaei P . Hematopoietic stem cell transplantation in adolescents and young adults. Acta Haematol. 2014;132(3–4):313–325. 10.1159/000360211 25228557

[7] Salchow J , Mann J , Koch B , von Grundherr J , Jensen W , Elmers S , et al. Comprehensive assessments and related interventions to enhance the long‐term outcomes of child, adolescent and young adult cancer survivors ‐ presentation of the CARE for CAYA‐program study protocol and associated literature review. BMC Cancer. 2020;20(1):16. 10.1186/s12885-019-6492-5 PMC6945396 31906955

[8] Sun CL , Francisco L , Kawashima T , Leisenring W , Robison LL , Baker KS , et al. Prevalence and predictors of chronic health conditions after hematopoietic cell transplantation: a report from the bone marrow transplant survivor study. Blood. 2010;116(17):3129–3139; quiz 377. 10.1182/blood-2009-06-229369 PMC2995350 20656930

[9] Lee CJ , Kim S , Tecca HR , Bo‐Subait S , Phelan R , Brazauskas R , et al. Late effects after ablative allogeneic stem cell transplantation for adolescent and young adult acute myeloid leukemia. Blood Adv. 2020;4(6):983–992. 10.1182/bloodadvances.2019001126 PMC7094022 32168378

[10] Ferry C , Gemayel G , Rocha V , Labopin M , Esperou H , Robin M , et al. Long‐term outcomes after allogeneic stem cell transplantation for children with hematological malignancies. Bone Marrow Transplant. 2007;40(3):219–224. 10.1038/sj.bmt.1705710 17530002

[11] Armenian SH , Sun CL , Kawashima T , Arora M , Leisenring W , Sklar CA , et al. Long‐term health‐related outcomes in survivors of childhood cancer treated with HSCT versus conventional therapy: a report from the bone marrow transplant survivor study (BMTSS) and childhood cancer survivor study (CCSS). Blood. 2011;118(5):1413–1420. 10.1182/blood-2011-01-331835 PMC3152502 21652685

[12] Wolff D , Herzberg PY , Herrmann A , Pavletic SZ , Heussner P , Mumm F , et al. Post‐transplant multimorbidity index and quality of life in patients with chronic graft‐versus‐host disease‐results from a joint evaluation of a prospective German multicenter validation trial and a cohort from the National Institutes of Health. Bone Marrow Transplant. 2021;56(1):243–256. 10.1038/s41409-020-01017-8 PMC8376641 32737446

[13] Charlson ME , Pompei P , Ales KL , MacKenzie CR . A new method of classifying prognostic comorbidity in longitudinal studies: development and validation. J Chronic Dis. 1987;40(5):373–383. 10.1016/0021-9681(87)90171-8 3558716

[14] Sorror ML , Maris MB , Storb R , Baron F , Sandmaier BM , Maloney DG , et al. Hematopoietic cell transplantation (HCT)‐specific comorbidity index: a new tool for risk assessment before allogeneic HCT. Blood. 2005;106(8):2912–2919. 10.1182/blood-2005-05-2004 PMC1895304 15994282

[15] Kahn J , Brazauskas R , Bo‐Subait S , Buchbinder D , Hamilton BK , Schoemans H , et al. Late effects after allogeneic haematopoietic cell transplantation in children and adolescents with non‐malignant disorders: a retrospective cohort study. Lancet Child Adolescent Health. 2024;8(10):740–750. 10.1016/S2352-4642(24)00167-6 PMC11588140 39217999

[16] Wilhelmsson M , Vatanen A , Borgstrom B , Gustafsson B , Taskinen M , Saarinen‐Pihkala UM , et al. Adverse health events and late mortality after pediatric allogeneic hematopoietic SCT‐two decades of longitudinal follow‐up. Bone Marrow Transplant. 2015;50(6):850–857. 10.1038/bmt.2015.43 25798676

[17] Tichelli A , Bucher C , Rovo A , Stussi G , Stern M , Paulussen M , et al. Premature cardiovascular disease after allogeneic hematopoietic stem‐cell transplantation. Blood. 2007;110(9):3463–3471. 10.1182/blood-2006-10-054080 17664354

[18] Chow EJ , Mueller BA , Baker KS , Cushing‐Haugen KL , Flowers ME , Martin PJ , et al. Cardiovascular hospitalizations and mortality among recipients of hematopoietic stem cell transplantation. Ann Intern Med. 2011;155(1):21–32. 10.7326/0003-4819-155-1-201107050-00004 21727290

[19] Neuhauser H , Kuhnert R , Born S . 12‐month prevalence of hypertension in Germany. J Health Monit. 2017;2(1):51–57. 10.17886/RKI-GBE-2017-016 PMC10161282 37151301

[20] Premstaller M , Perren M , Koçack K , Arranto C , Favre G , Lohri A , et al. Dyslipidemia and lipid‐lowering treatment in a hematopoietic stem cell transplant cohort: 25 years of follow‐up data. J Clin Lipidol. 2018;12(2):464–480.e3. 10.1016/j.jacl.2017.11.008 29310991

[21] Murnane A , Gough K , Thompson K , Holland L , Conyers R . Adolescents and young adult cancer survivors: exercise habits, quality of life and physical activity preferences. Support Care Cancer. 2015;23(2):501–510. 10.1007/s00520-014-2446-2 25294658

[22] Annaloro C , Usardi P , Airaghi L , Giunta V , Forti S , Orsatti A , et al. Prevalence of metabolic syndrome in long‐term survivors of hematopoietic stem cell transplantation. Bone Marrow Transplant. 2008;41(9):797–804. 10.1038/sj.bmt.1705972 18195686

[23] Kirsch M , Gotz A , Halter JP , Schanz U , Stussi G , Dobbels F , et al. Differences in health behaviour between recipients of allogeneic haematopoietic SCT and the general population: a matched control study. Bone Marrow Transplant. 2014;49(9):1223–1230. 10.1038/bmt.2014.142 25046214

[24] Majhail NS , Lazarus HM , Burns LJ . Iron overload in hematopoietic cell transplantation. Bone Marrow Transplant. 2008;41(12):997–1003. 10.1038/bmt.2008.99 18438425

[25] Bergeron A , Chevret S , Peffault de Latour R , Chagnon K , de Margerie‐Mellon C , Riviere F , et al. Noninfectious lung complications after allogeneic haematopoietic stem cell transplantation. Eur Respir J. 2018;51(5):1702617. 10.1183/13993003.02617-2017 29650555

[26] Rizzo JD , Wingard JR , Tichelli A , Lee SJ , Van Lint MT , Burns LJ , et al. Recommended screening and preventive practices for long‐term survivors after hematopoietic cell transplantation: joint recommendations of the European Group for Blood and Marrow Transplantation, Center for International Blood and Marrow Transplant Research, and the American Society for Blood and Marrow Transplantation (EBMT/CIBMTR/ASBMT). Bone Marrow Transplant. 2006;37(3):249–261. 10.1038/sj.bmt.1705243 16435004

[27] Loren AW , Senapati S . Fertility preservation in patients with hematologic malignancies and recipients of hematopoietic cell transplants. Blood. 2019;134(9):746–760. 10.1182/blood.2018846790 31292116

[28] Bilic E , Delimar V , Desnica L , Pulanic D , Bilic E , Bakovic M , et al. High prevalence of small‐ and large‐fiber neuropathy in a prospective cohort of patients with moderate to severe chronic GvHD. Bone Marrow Transplant. 2016;51(11):1513–1517. 10.1038/bmt.2016.158 PMC6787914 27272447

[29] Shiota T , Eura N , Hasegawa A , Kiriyama T , Sugie K . Pathological features of inflammatory myopathy as a manifestation of chronic graft‐versus‐host disease after allogeneic bone marrow transplantation. Neuropathology. 2022;42(4):309–314. 10.1111/neup.12816 35508303

[30] Di Giuseppe G , Thacker N , Schechter T , Pole JD . Anxiety, depression, and mental health‐related quality of life in survivors of pediatric allogeneic hematopoietic stem cell transplantation: a systematic review. Bone Marrow Transplant. 2020;55(7):1240–1254. 10.1038/s41409-020-0782-z 31980743

[31] Cooke L , Chung C , Grant M . Psychosocial care for adolescent and young adult hematopoietic cell transplant patients. J Psychosoc Oncol. 2011;29(4):394–414. PMC3268701 21966725

[32] Pulewka K , Wolff D , Herzberg PY , Greinix H , Heussner P , Mumm FHA , et al. Physical and psychosocial aspects of adolescent and young adults after allogeneic hematopoietic stem‐cell transplantation: results from a prospective multicenter trial. J Cancer Res Clin Oncol. 2017;143(8):1613–1619. 10.1007/s00432-017-2424-4 PMC11819110 28424902

[33] Hautmann AH , Elad S , Lawitschka A , Greinix H , Bertz H , Halter J , et al. Metabolic bone diseases in patients after allogeneic hematopoietic stem cell transplantation: report from the consensus conference on clinical practice in chronic graft‐versus‐host disease. Transpl Int. 2011;24(9):867–879. 10.1111/j.1432-2277.2011.01264.x 21557780

[34] Yang Y‐M , Guo S‐J , Xiao R , Yu X‐J , Liu Y‐P , Shuai P . Prevalence of osteoporosis among patients after stem cell transplantation: a systematic review and meta‐analysis. Bone Marrow Transplant. 2024;59(6):785–794. 10.1038/s41409-024-02243-0 38424174

[35] Saddadi F , Hakemi M , Najafi I , Moghadam K , Ghavamzadeh A , Jahani M , et al. Chronic kidney disease after hematopoietic cell transplantation: frequency, risk factors, and outcomes. Transplant Proc. 2009;41(7):2895–2897. 10.1016/j.transproceed.2009.07.093 19765466

[36] Ellis MJ , Parikh CR , Inrig JK , Kanbay M , Patel UD . Chronic kidney disease after hematopoietic cell transplantation: a systematic review. Am J Transplant. 2008;8(11):2378–2390. 10.1111/j.1600-6143.2008.02408.x PMC3564956 18925905

[37] Ogonek J , Kralj Juric M , Ghimire S , Varanasi PR , Holler E , Greinix H , et al. Immune reconstitution after allogeneic hematopoietic stem cell transplantation. Front Immunol. 2016;7:507. 10.3389/fimmu.2016.00507 PMC5112259 27909435

[38] Danylesko I , Shimoni A . Second malignancies after hematopoietic stem cell transplantation. Curr Treat Options Oncol. 2018;19(2):9. 10.1007/s11864-018-0528-y 29423555

[39] Cooke KR , Luznik L , Sarantopoulos S , Hakim FT , Jagasia M , Fowler DH , et al. The biology of chronic graft‐versus‐host disease: a task force report from the National Institutes of Health consensus development project on criteria for clinical trials in chronic graft‐versus‐host disease. Biol Blood Marrow Transplant. 2017;23(2):211–234. 10.1016/j.bbmt.2016.09.023 PMC6020045 27713092

[40] Bachier CR , Aggarwal SK , Hennegan K , Milgroom A , Francis K , Dehipawala S , et al. Epidemiology and treatment of chronic graft‐versus‐host disease post‐allogeneic hematopoietic cell transplantation: a US claims analysis. Transplant Cell Ther. 2021;27(6):504.e1–504.e6. 10.1016/j.jtct.2020.12.027 34158154

[41] Grain A , Rialland‐Battisti F , Chevallier P , Blin N , Dalle JH , Michel G , et al. Hematopoietic stem cell transplantation for acute lymphoblastic leukemia: why do adolescents and young adults outcomes differ from those of children? A retrospective study on behalf of the francophone Society of Stem Cell Transplantation and Cellular Therapy (SFGM‐TC). J Cancer Res Clin Oncol. 2022;149:1473–1483. 10.1007/s00432-022-04021-1 PMC11797360 35507103

