# Supplementary material for: Comorbid conditions after allogeneic haematopoietic stem cell transplantation in young adults in Germany—A prospective, multicentre trial
Source: Br J Haematol. 2026 Aug 10;209(4):1807–17. doi: 10.1111/bjh.70738 (PMC13674112; doi:10.1111/bjh.70738)
Supplement: Supplementary file 1 [file BJH-209-1807-s001.docx]

**Supplementary Information table S2: determinants of single PTMI-defined comorbidities**

|  |  | **Hypogonadism** | **Infection** | **Liver condition** | **Lung impairment** | **Osteopenia/**  **osteoporosis** | **Hypo-**  **thyroi-**  **dism** | **Dyslipidemia** | **Renal impairment** | **Neurologic**  **condition** | **Psychiatric**  **disorder** | **Avascular necrosis** | **Nicotine abuse** | **Obesity** |
| --- | --- | --- | --- | --- | --- | --- | --- | --- | --- | --- | --- | --- | --- | --- |
| **TBI** | **d100** | p=0.575 |  |  | p=0.777 | p=0.471 | p=0.729 | p=0.849 | p=0.235 | p=0.106 |  | p=0.252 |  |  |
|  | **d180** | p=0.309 |  |  | p=0.755 | p=0.601 | p=0.588 | p=0.240 | p=0.157 | p=1.000 |  | p=0.261 |  |  |
|  | **d365** | p=0.560 |  |  | p=0.694 | p=0.078 | p=0.394 | p=0.086 | p=1.000 | p=0.282 |  | p=0.138 |  |  |
|  | **d910** | p=0.056 |  |  | p=0.088 | p=0.696 | p=0.362 | p=0.145 | p=0.678 | p=0.305 |  | p=0.436 |  |  |
| **MAC** | **d100** | p=0.232 |  |  |  |  | p=0.546 | p=0.235 | **p=0.047 (Phi=-0.217)** | **p=0.011 (Phi=0.262)** |  |  |  |  |
|  | **d180** | p=0.266 |  |  |  |  | p=0.772 | p=0.907 | **p=0.044(Phi=-0.238)** | p=0.534 |  |  |  |  |
|  | **d365** | p=0.165 |  |  |  |  | p=0.168 | p=0.458 | p=0.256 | p=0.531 |  |  |  |  |
|  | **d910** | p=0.945 |  |  |  |  | p=0.645 | p=0.969 | p=0.652 | p=0.726 |  |  |  |  |
| **Chronic GvHD** | **d100** | **p=0.001 (Phi=0.389)** | p=0.512 | p=0.684 | p=0.192 | p=1.000 | p=0.608 | p=0.684 | p=1.000 | p=0.713 |  | p=1.000 |  |  |
|  | **d180** | p=0.256 | p=0.858 | p=0.218 | p=1.000 | p=0.102 | p=1.000 | p=0.235 | p=0.316 | p=0.496 |  | **p=0.021 (Phi=0.298)** |  |  |
|  | **d365** | p=0.054 | p=0.322 | p=0.134 | p=1.000 | p=0.566 | p=0.167 | p=1.000 | p=1.000 | p=1.000 |  | p=1.000 |  |  |
|  | **d910** | p=1.000 | p=1.000 | p=1.000 | p=0.576 | **p=0.022 (Phi=0.371)** | p=1.000 | p=0.661 | p=0.548 | p=1.000 |  | p=1.000 |  |  |
| **Iron overload** | **d100** |  |  | **p=0.023 (Phi=0.234)** |  |  |  |  |  |  |  |  |  |  |
|  | **d180** |  |  | **p=0.016 (Phi=0.258)** |  |  |  |  |  |  |  |  |  |  |
|  | **d365** |  |  | p=0.217 |  |  |  |  |  |  |  |  |  |  |
|  | **d910** |  |  | **p=0.002 (Phi=0.436)** |  |  |  |  |  |  |  |  |  |  |
| **Infection** | **d100** |  |  |  | p=0.120 |  |  |  |  |  |  |  | **p=0.035 (Phi=0.249)** |  |
|  | **d180** |  |  |  | **p=0.007 (Phi= 0.287)** |  |  |  |  |  |  |  | p=1.000 |  |
|  | **d365** |  |  |  | p=0.774 |  |  |  |  |  |  |  | p=0.204 |  |
|  | **d910** |  |  |  | p=0.783 |  |  |  |  |  |  |  | p=1.000 |  |
| **Gender**  **of the**  **patient** | **d100** | **p=0.010 (Phi=0.263)** |  |  |  | p=0.061 | p=0.151 | p=0.325 |  |  | p=1.000 | p=0.555 |  | p=0.737 |
|  | **d180** | **p=0.008 (Phi=0.281)** |  |  |  | p=0.341 | p=0.433 | p=0.427 |  |  | p=1.000 | p=1.000 |  | p=0.730 |
|  | **d365** | **p=0.003 (Phi=0.334)** |  |  |  | p=0.308 | p=0.964 | p=0.671 |  |  | p=0.302 | p=0.641 |  | p=0.644 |
|  | **d910** | **p=0.044 (Phi=0.283)** |  |  |  | p=0.167 | p=0.391 | p=0.431 |  |  | p=0.214 | p=0.427 |  | p=1.000 |
| **Ongoing immuno-suppression** | **d100** | p=0.815 | p=0.571 | p=1.000 | p=1.000 | p=1.000 | p=1.000 | p=1.000 | p=1.000 | p=0.542 |  | p=1.000 |  |  |
|  | **d180** | p=0.857 | p=0.213 | p=0.494 | p=1.000 | p=0.785 | p=0.134 | p=0.268 | p=1.000 | p=0.246 |  | p=0.245 |  |  |
|  | **d365** | p=0.287 | p=0.289 | p=0.671 | p=0.419 | p=0.069 | p=0.679 | p=0.463 | p=0.437 | p=1.000 |  | **p=0.020 (Phi=-0.299)** |  |  |
|  | **d910** | p=1.000 | p=0.571 | p=1.000 | p=0.309 | **P=0.021 (Phi=-0.391)** | p=1.000 | p=0.602 | p=1.000 | p=1.000 |  | p=0.432 |  |  |
| **Arterial**  **hypertension** | **d100** |  |  |  |  |  |  | p=0.686 | p=0.186 |  |  |  |  |  |
|  | **d180** |  |  |  |  |  |  | p=0.553 | p=01.000 |  |  |  |  |  |
|  | **d365** |  |  |  |  |  |  | p=0.763 | **p=0.026 (Phi= 0.288)** |  |  |  |  |  |
|  | **d910** |  |  |  |  |  |  | p=0.327 | **p=0.017 (Phi=0.433)** |  |  |  |  |  |
| **Hypogonadism** | **d100** |  |  |  |  | **p=0.017 (Phi=0.244)** |  |  |  |  |  |  |  |  |
|  | **d180** |  |  |  |  | p=0.121 |  |  |  |  |  |  |  |  |
|  | **d365** |  |  |  |  | p=0.606 |  |  |  |  |  |  |  |  |
|  | **d910** |  |  |  |  | p=0.696 |  |  |  |  |  |  |  |  |
| **Obesity** | **d100** |  |  |  |  |  |  | p=0.458 |  |  |  |  | p=0.558 |  |
|  | **d180** |  |  |  |  |  |  | p=1.000 |  |  |  |  | p=0.487 |  |
|  | **d365** |  |  |  |  |  |  | p=1.000 |  |  |  |  | p=1.000 |  |
|  | **d910** |  |  |  |  |  |  | p=0.058 |  |  |  |  | p=1.000 |  |
| **Avascular**  **necrosis** | **d100** |  |  |  |  |  |  | p=0.336 |  |  |  |  |  |  |
|  | **d180** |  |  |  |  |  |  | p=0.479 |  |  |  |  |  |  |
|  | **d365** |  |  |  |  |  |  | p=1.000 |  |  |  |  |  |  |
|  | **d910** |  |  |  |  |  |  | p=0.376 |  |  |  |  |  |  |

GvHD: graft-versus-host disease, MAC: myeloablative conditioning, TBI: total body irradiation
