# Supplementary material for: Comorbid conditions after allogeneic haematopoietic stem cell transplantation in young adults in Germany—A prospective, multicentre trial
Source: Br J Haematol. 2026 Aug 10;209(4):1807–17. doi: 10.1111/bjh.70738 (PMC13674112; doi:10.1111/bjh.70738)
Supplement: Supplementary file 2 [file BJH-209-1807-s002.docx]

**Supplementary Table S3: Frequency of comorbidities captured by the PTMI including the applied definition**

|  |  | **day 100 (n = 95)** | | **day 180 (n = 88)** | | **day 365 (n = 82)** | | **day 910 (n = 51)** | |
| --- | --- | --- | --- | --- | --- | --- | --- | --- | --- |
| **Comorbidity (n = number; % = frequency)** | **Definition** | **n** | **%** | **n** | **%** | **n** | **%** | **n** | **%** |
| **1. Cardiovascular** |  |  |  |  |  |  |  |  |  |
| Arrhythmia | Atrial fibrillation or flutter, sick sinus syndrome or ventricular arrhythmias | 2 | 2.1% | 1 | 1.1% | 0 | 0.0% | 1 | 2.0% |
| Coronary artery disease | Coronary artery disease (one or more vessel-coronary artery stenosis requiring medical treatment, stent, or bypass graft) | 0 | 0.0% | 0 | 0.0% | 0 | 0.0% | 0 | 0.0% |
| History of MI | History of myocardial infarction | 0 | 0.0% | 0 | 0.0% | 0 | 0.0% | 0 | 0.0% |
| Heart valve disease | Except asymptomatic mitral valve prolapse and trace/physiologic regurgitation on echocardiogram | 1 | 1.1% | 1 | 1.1% | 1 | 1.2% | 1 | 2.0% |
| Congestive heart failure: Mild/moderate | EF 30-50% | 1 | 1.1% | 2 | 2.3% | 4 | 4.9% | 1 | 2.0% |
| Congestive heart failure: Severe | EF<30% | 0 | 0.0% | 0 | 0.0% | 0 | 0.0% | 0 | 0.0% |
| Cerebrovascular disease | Transient ischemic attack or history of cerebrovascular accident, or neurologic impairment consequent to CVA | 2 | 2.1% | 1 | 1.1% | 2 | 2.4% | 0 | 0.0% |
| Peripheral vascular disease | Resulting in impairment of daily activities and requiring pharmacologic treatment | 0 | 0.0% | 0 | 0.0% | 0 | 0.0% | 0 | 0.0% |
| Venous thrombosis | Confirmed by radiographic/ultrasound testing and requiring anticoagulation | 9 | 9.5% | 7 | 8.0% | 2 | 2.4% | 0 | 0.0% |
| Compensated hypertension | Receiving an antihypertensive agent for treatment of hypertension | 27 | 28.4% | 25 | 28.4% | 14 | 17.1% | 6 | 11.8% |
| Uncontrolled hypertension | Repeated blood pressure measurements greater than 160 systolic or greater than 100 diastolic, despite pharmacologic intervention | 1 | 1.1% | 0 | 0.0% | 0 | 0.0% | 0 | 0.0% |
| **2. Gastrointestinal** |  |  |  |  |  |  |  |  |  |
| Peptic ulcer/hernia/reflux | Requiring treatment | 2 | 2.1% | 1 | 1.1% | 0 | 0.0% | 0 | 0.0% |
| Mild hepatic | Chronic hepatitis, bilirubin >ULN to 1.5 x ULN, or AST/ALT>ULN to 2.5 x ULN | 9 | 9.5% | 8 | 9.1% | 5 | 6.1% | 2 | 3.9% |
| Moderate/severe hepatic | Liver cirrhosis, bilirubin >1.5 times ULN or AST/ALT> 2.5 x ULN | 2 | 2.1% | 3 | 3.4% | 1 | 1.2% | 0 | 0.0% |
| Iron overload | Ferritin > 1000µg/ml | 47 | 49.5% | 42 | 47.7% | 35 | 42.7% | 9 | 17.6% |
| Hepatitis B | Hep B Surface-Antigen positive and/or PCR positive or receiving treatment for Hep B | 0 | 0.0% | 0 | 0.0% | 0 | 0.0% | 0 | 0.0% |
| Hepatitis C | PCR positive or receiving treatment for Hep C | 0 | 0.0% | 0 | 0.0% | 0 | 0.0% | 0 | 0.0% |
| Inflammatory bowel disease | Crohn’s disease or ulcerative colitis prior to transplant | 1 | 1.1% | 0 | 0.0% | 0 | 0.0% | 0 | 0.0% |
| **3. Pulmonary** |  |  |  |  |  |  |  |  |  |
| Moderate pulmonary | DLCO and/or FEV-1 66% - 80% with dyspnea on slight activity | 8 | 8.4% | 11 | 12.5% | 6 | 7.3% | 6 | 11.8% |
| Severe pulmonary | DLCO and/or FEV-1 65% or lower with dyspnea at rest or requiring oxygen | 2 | 2.1% | 0 | 0.0% | 1 | 1.2% | 1 | 2.0% |
| Pulmonary Hypertension | Mean pulmonary artery pressure in rest >25mm Hg or systolic pulmonary artery pressure at rest >40mmHg | 0 | 0.0% | 0 | 0.0% | 0 | 0.0% | 1 | 2.0% |
| Asthma | Asthma symptoms for which inhaled steroids or other daily treatments are needed chronically to prevent or manage attacks and/or reversible obstruction on pulmonary function studies | 0 | 0.0% | 1 | 1.1% | 1 | 1.2% | 1 | 2.0% |
| **4. Endocrine** |  |  |  |  |  |  |  |  |  |
| Hyperglycemia | Fasting blood sugar greater than ≥126 mg/dl or random blood sugar greater than 200 mg/dl | 0 | 0.0% | 1 | 1.1% | 1 | 1.2% | 1 | 2.0% |
| Diabetes | Requiring treatment with insulin or oral hypoglycemic agents but not diet alone | 5 | 5.3% | 2 | 2.3% | 1 | 1.2% | 1 | 2.0% |
| Hypothyroidism | Including compensated hypothyroidism | 8 | 8.4% | 10 | 11.4% | 13 | 15.9% | 5 | 9.8% |
| Hyperthyroidism | Suppressed TSH not associated with thyroid hormone over-replacement | 0 | 0.0% | 3 | 3.4% | 0 | 0.0% | 1 | 2.0% |
| Hypogonadism | Women who are post-menopausal (at least 6 months without menses), need for testosterone replacement or free testostorone below the lower limit | 30 | 31.6% | 34 | 38.6% | 32 | 39.0% | 23 | 45.1% |
| Adrenal Insufficiency | Including compensated adrenal insufficiency | 3 | 3.2% | 2 | 2.3% | 2 | 2.4% | 1 | 2.0% |
| Hypertriglyceridemia | Requiring treatment or above 2 times the upper normal limit | 7 | 7.4% | 7 | 8.0% | 14 | 17.1% | 7 | 13.7% |
| Hypercholesterolemia | Requiring treatment or total cholesterol greater than 1.5 times the upper limit of normal value or LDL cholesterol > 160 mg/dl | 8 | 8.4% | 14 | 15.9% | 13 | 15.9% | 11 | 21.6% |
| **5. Neuropsychiatric** |  |  |  |  |  |  |  |  |  |
| Psychiatric disturbance-Depression | Depression requiring psychiatric consult or treatment for depression | 3 | 3.2% | 1 | 1.1% | 4 | 4.9% | 0 | 0.0% |
| Psychiatric disturbance-Anxiety or panic disorder | Anxiety or panic disorder requiring psychiatric consult or treatment for anxiety | 5 | 5.3% | 4 | 4.5% | 5 | 6.1% | 6 | 11.8% |
| Dementia | Presence of neurocognitive impairment including memory, attention, language, and problem solving persisting for at least 6 months | 0 | 0.0% | 0 | 0.0% | 0 | 0.0% | 0 | 0.0% |
| Neurologic disease (peripheral neuropathy, MS, Parkinson’s disease or other chronic neurologic disease) | Symptomatic and requiring treatment to control or manage symptoms/disease process | 9 | 9.5% | 8 | 9.1% | 5 | 6.1% | 5 | 9.8% |
| Visual impairment secondary to cataracts, glaucoma or macular degeneration | Unilateral or bilateral, and unrepaired | 2 | 2.1% | 1 | 1.1% | 1 | 1.2% | 3 | 5.9% |
| Hearing impairment | Unilateral or bilateral, and unrepaired | 1 | 1.1% | 0 | 0.0% | 1 | 1.2% | 0 | 0.0% |
| **6. Bone/Joint** |  |  |  |  |  |  |  |  |  |
| Osteoarthritis | Symptomatic and requiring treatment, or symptomatic and with osteoarthritic changes noted on radiographic studies | 0 | 0.0% | 1 | 1.1% | 0 | 0.0% | 5 | 9.8% |
| Degenerative disc disease (spinal stenosis or severe chronic back pain) | Symptomatic and requiring treatment or symptomatic and with degenerative disc disease noted on radiographic studies | 0 | 0.0% | 0 | 0.0% | 1 | 1.2% | 0 | 0.0% |
| Avascular necrosis | Symptomatic with pain secondary to AVN or joint replacement | 3 | 3.2% | 3 | 3.4% | 4 | 4.9% | 7 | 13.7% |
| Osteopenia/Osteoporosis | T Score < or equal to minus 1.5 or on treatment with a bisphosphonate (not as part of treatment for MM) | 9 | 9.5% | 14 | 15.9% | 23 | 28.0% | 12 | 23.5% |
| Rheumatologic | Lupus, mixed connective tissue disorder, rheumatoid arthritis (confirmed serological and/or by typical Xray, polymyalgia rheumatica | 0 | 0.0% | 1 | 1.1% | 1 | 1.2% | 1 | 2.0% |
| **7. Other comorbidities** |  |  |  |  |  |  |  |  |  |
| Mild renal | Serum creatinine >1.2 mg/dl but less than 2 | 7 | 7.4% | 5 | 5.7% | 6 | 7.3% | 5 | 9.8% |
| Moderate/severe renal | Serum creatinine ≥2 mg/dL, on dialysis, or prior renal transplantation | 0 | 0.0% | 0 | 0.0% | 2 | 2.4% | 1 | 2.0% |
| Proteinuria | >1 gram/day or 1 g/g Creatinine | 1 | 1.1% | 1 | 1.1% | 2 | 2.4% | 1 | 2.0% |
| Prior solid malignancy | Treated at any time point in the patient’s past history before transplantation, excluding nonmelanoma skin cancer | 4 | 4.2% | 4 | 4.5% | 5 | 6.1% | 3 | 5.9% |
| Solid malignancy | Occurrence of a solid malignancy (excluding nonmelanoma skin cancer) after transplantation | 2 | 2.1% | 2 | 2.3% | 1 | 1.2% | 0 | 0.0% |
| Nonmelanoma skin cancer | Occurrence of a non-melanoma skin cancer after transplantation | 0 | 0.0% | 0 | 0.0% | 0 | 0.0% | 0 | 0.0% |
| Post-transplant lymphoproliferative disorder (PTLD) | Occurrence of a PTLD after transplantation | 2 | 2.1% | 0 | 0.0% | 0 | 0.0% | 0 | 0.0% |
| Nicotine abuse | Daily use/consumption of any substance containing nicotine | 7 | 7.4% | 6 | 6.8% | 5 | 6.1% | 1 | 2.0% |
| Mild infection | Any mild infection which has been treated as outpatient or did not require hospitalization for itself (oral HSV or candida, oral treatment for bacterial infection (without pneumonia), asymptomatic CMV reactivation | 15 | 15.8% | 12 | 13.6% | 15 | 18.3% | 10 | 19.6% |
| Moderate infection | Infection requiring hospitalization for supportive care/treatment, or intravenous antibiotics or CMV/VZV with fever only, or fungal pneumonia without respiratory/constitutional symptoms. | 5 | 5.3% | 8 | 9.1% | 2 | 2.4% | 2 | 3.9% |
| Severe infection | Admitted for treatment and with life threatening manifestations/consequences of infection (eg. severe sepsis, organ complications, aspergillosis- associated organ destruction/invasion, viral encephalitis, severe varicella zoster with organ complications such as pneumonia, zoster ophthalmicus, hepatitis, CMV with enteritis, hepatitis, or pneumonia; PCP infection with respiratory symptoms, toxoplasmosis with organ involvement, nocardia infection) | 1 | 1.1% | 1 | 1.1% | 1 | 1.2% | 1 | 2.0% |
| Obesity | body mass index >30 and <35 (weight in kg/height in meters^2^) | 8 | 8.4% | 6 | 6.8% | 3 | 3.7% | 3 | 5.9% |
| Severe obesity | Obesity and/or body mass index ≥35 (weight in kg/height in meters^2^) | 2 | 2.1% | 3 | 3.4% | 2 | 2.4% | 1 | 2.0% |
| Underweight | Body mass index <18,5 (kg/m²) | 11 | 11.6% | 9 | 10.2% | 4 | 4.9% | 1 | 2.0% |
| Other comorbid conditions (patients affected) | comorbid conditions present but not captured above | 5 (5) | 5.3% | 6 (6) | 6.8% | 9 (6) | 8.5% | 8 (6) | 15.7% |
