# Supplementary material for: Comorbid conditions after allogeneic haematopoietic stem cell transplantation in young adults in Germany—A prospective, multicentre trial
Source: Br J Haematol. 2026 Aug 10;209(4):1807–17. doi: 10.1111/bjh.70738 (PMC13674112; doi:10.1111/bjh.70738)
Supplement: Supplementary file 3 [file BJH-209-1807-s003.docx]

**Supplementary Information S1: Questionnaire of patient-reported comorbidities**

**Cardiovascular Diseases**

## Do you suffer from any of the following conditions?

## High blood pressure: ❒ yes ❒ no

## Heart attack: ❒ yes ❒ no If yes, when did it occur? _____

## Stroke: ❒ yes ❒ no If yes, when did it occur? _____

## Cardiac arrhythmia: ❒ yes ❒ no

## Heart failure: ❒ yes ❒ no

## Are you aware of any disease of the heart valves? ❒ yes ❒ no

**Respiratory Diseases**

Do you suffer from shortness of breath?

during high exertion	❒ yes	❒ no

during mild exertion	❒ yes	❒ no

at rest	❒ yes	❒ no

Do you suffer from frequent respiratory infections?	❒ yes	❒ no

If you answered „yes“ to the previous question, how often per year? _________

**Gastrointestinal Diseases, Liver Diseases**

Have you been diagnosed with iron overload?	❒ yes	❒ no

## Have you been diagnosed with liver inflammation (hepatitis)? ❒ yes ❒ no

## Did any other gastrointestinal diseases occur? ❒ yes ❒ no

If yes, which ones? _________________________________________________

**Kidney diseases**

## Have you developed a kidney disease after the transplantation? ❒ yes ❒ no

## Did you require Dialysis? ❒ yes ❒ no

**Metabolic disorders**

## Have you been diagnosed with a blood sugar disorder (diabetes mellitus)? ❒ yes ❒ no

## Have you been diagnosed with elevated blood lipid levels (e.g. cholesterol)? ❒ yes ❒ no

## Do you suffer from an underactive thyroid (hypothyroidism)? ❒ yes ❒ no

## Do you suffer from an overactive thyroid (hyperthyroidism)? ❒ yes ❒ no

## Are you receiving hormone replacement therapy? ❒ yes ❒ no

## **Eye diseases**

## Has your vision worsened after the transplantation? ❒ yes ❒ no

## Do you suffer or did you suffer from clouding of the eye lens (cataract)? ❒ yes ❒ no

## If yes, was surgical treatment required? ❒ yes ❒ no

## Do you suffer from dry eyes? ❒ yes ❒ no

**Disorders of the Skeletal System**

## Do you suffer from - Bone loss (osteoporosis) ❒ yes ❒ no

## - Bone infarction (osteonecrosis) ❒ yes ❒ no

## Have you required an artificial joint replacement? ❒ yes ❒ no

## Which joint must/needed to be replaced? ___________ When was this performed? _________

**Nervous System**

Do you suffer from symptoms such as:

## - Memory problems, forgetfulness ❒ yes ❒ no

## - Concentration difficulties ❒ yes ❒ no

## - Tingling or numbness? ❒ yes ❒ no

**Cancer**

## Have any additional cancers occurred after the transplantation? ❒ yes ❒ no

If yes, when and which ones? _______________________________________________________

**Other**

## Do you suffer from any other illnesses or complaints? ❒ yes ❒ no

If yes, which ones? ________________________________________________________________
